# Effectiveness of Suprascapular Nerve Block in the Treatment of Hemiplegic Shoulder Pain: A Systematic Review and Meta-Analysis

**DOI:** 10.3389/fneur.2021.723664

**Published:** 2021-10-05

**Authors:** Yajing Hou, Yong Wang, Xiaojing Sun, Yake Lou, Ying Yu, Tong Zhang

**Affiliations:** ^1^School of Rehabilitation Medicine, Capital Medical University, Department of Neurological Rehabilitation, Beijing Bo'ai Hospital, China Rehabilitation Research Center, Lab of Brain Injury Repair and Rehabilitation, China Rehabilitation Science Institute, Beijing, China; ^2^Rehabilitation Medicine Center, Fuxing Hospital, Capital Medical University, Beijing, China; ^3^Capital Medical University, Beijing Rehabilitation Hospital, Shijingshan, Beijing, China; ^4^Department of Cardiology, Beijing Anzhen Hospital, Beijing Institute of Heart Lung and Blood Vessel Disease, Capital Medical University, Beijing, China; ^5^Department of Neurology, Beijing Tiantan Hospital, Capital Medical University, Beijing, China

**Keywords:** suprascapular nerve block, hemiplegic shoulder pain, meta-analysis, nerve block, shoulder pain, systematic review

## Abstract

**Purpose:** We aimed to investigate the effectiveness of suprascapular nerve block (SSNB) in patients with hemiplegic shoulder pain (HSP).

**Background:** SSNB is widely used in various shoulder pains, but whether it is effective in HSP remains unknown.

**Methods:** PubMed, Cochrane Library, and Embase databases were searched to identify potential citations. Randomized controlled trials meeting the eligible criteria were included in our analysis. The primary endpoint was Visual Analog Scale (VAS) with a maximum value of 100 and a minimum value of 0. Secondary endpoints were passive range of motion (PROM) that pain starts, and the PROM mainly included abduction, flexion, and external rotation. In addition, the upper extremity Fugl-Meyer assessment (FMA) was also included in our secondary endpoints.

**Results:** Eight studies with 281 patients were included in our analysis. For VAS, there was no obvious difference between SSNB group and control group regardless of the follow-up period (<4 weeks or ≥4 weeks), which were −6.62 (−15.76, 2.53; *p* = 0.16) and 1.78 (−16.18, 19.74; *p* = 0.85). For shoulder function, the PROM of abduction, flexion, and external rotation was similar between groups. However, motor function indicator FMA is lower in SSNB control than that in control group, with a mean difference (and 95% CI) of −2.59 (−4.52, −0.66; *p* = 0.008).

**Conclusion:** SSNB is an effective way for HSP patients.

**Systematic Review Registration:** Registration ID: CRD42021252429.

## Introduction

Hemiplegic shoulder pain (HSP), as a very common poststroke complication, often occurs within a week after stroke (1). According to different studies, the incidence of HSP ranges from 16% to 84% in poststroke patients (2, 3). HSP patients may have nocturnal pain, but the most obvious pain is during passive external rotation and shoulder abduction (4), which limits the motion of the shoulder. In turn, the limited shoulder aggravates the HSP (5). As far as we know, the etiology of HSP is complex and varied, mainly including soft tissue lesions, muscle tone changes, and altered central nervous system phenomena (6). Currently, suprascapular nerve block (SSNB), botulinum toxin A, and intra-articular steroid injection are used in clinical practice, the optimal treatment for HSP still unknown (5).

About 70% of the shoulder joint sensorial fibers run through the suprascapular nerve (SSN) (7), so the blockage or damage of SSN may contribute to alleviating HSP. In recent years, some studies found that SSNB can reduce the pain intensity of HSP, thus improving the motion of shoulder joint (8, 9), but some other studies drew a negative conclusion that there is no difference in pain relief in a 6-week follow-up of SSNB for HSP (10); a study even found that SSNB is inferior to other treatments (11). Given the controversial effect of SSNB on HSP and the small samples in each study, it is important for us to perform a meta-analysis to investigate the real effects of SSNB in the treatment of HSP.

## Methods

### Search Strategy

The keywords “hemiplegia,” “monoplegia,” “paresis,” “spastic paresis,” “cerebrovascular accident,” “stroke,” “basal ganglia hemorrhage,” “brain ischemia,” “brain infarction,” “intracranial hemorrhage,” “subarachnoid hemorrhage,” “post-stroke,” “shoulder pain,” “suprascapular nerve block,” “blockade,” and “suprascapular fossa” were used to search Pubmed, Embase, and Cochrane database to identify potential randomized controlled trials (RCTs) until May 2, 2021 (further details are available in the Supplementary Material). Only citations whose titles and abstracts are published in English are potential for eligibility.

### Eligibility Criteria

Studies with the following criteria could be eligible for inclusion:

1: RCTs.
2: The intervention group is conducted with SSNB and the control group with placebo or active control.
3: At least one interesting outcome reported.
4: Sample size is not less than 10.

### Exclusion Criteria

1: Animal experiments.
2: Retrospective studies.
3: Cohort studies.
4: Studies with no randomization.

### Data Extraction and Quality Assessment

Two authors (YH and YW) independently screened the searched citations to find out eligible citations. Disagreement between YH and YW was resolved by another author (TZ). Any potential citations that were uncertain to meet the inclusion criteria would be evaluated further by reading the full text. After screening, YH and YW continued to extract data in the inclusion reference. Baseline characteristics, inclusion criteria, exclusion criteria, intervention measure, outcome measure, and results would be extracted independently by YH and YW. Quality assessment of included references was performed by YL and YY according to the Cochrane Handbook for Systematic Reviews of Interventions (version 5.1.0), and the main evaluation criteria included random sequence generation (selection bias), allocation concealment (selection bias), blinding of participants and personnel (performance bias), blinding of outcome assessment (detection bias), incomplete outcome data (attrition bias), selective reporting (reporting bias), and other biases.

### Outcomes

The primary endpoint for the present meta-analysis was Visual Analog Scale (VAS) with a maximum value of 100 and a minimum value of 0. Secondary endpoints were passive range of motion (PROM) that pain starts, and the PROM mainly included abduction, flexion, and external rotation. In addition, the upper extremity Fugl-Meyer assessment (FMA) was also included in our secondary endpoints.

### Statistical Analysis

All the statistical analyses were conducted using Review Manager (RevMan) version 5.3 (The Cochrane Collaboration, Copenhagen, Denmark) and Stata 15.1 (StataCorp, College Station, TX, USA) software. The study was performed in reference to the Preferred Reporting Items for Systematic reviews and Meta-Analyses (PRISMA) statement and was registered in the PROSPERO database (No.: CRD42021252429) (12). The mean difference and 95% confidence interval (CI) were calculated by inverse variance analysis. Considering the different control groups, we used the random-effects model to deal with possible heterogeneity. Sensitivity analysis was performed by sequentially omitting one trial, and publication bias was evaluated by the visual funnel plot. Subgroup analyses based on different controls were performed to detect the real effects of SSNB.

## Results

### Study Selection

**Figure 1:**
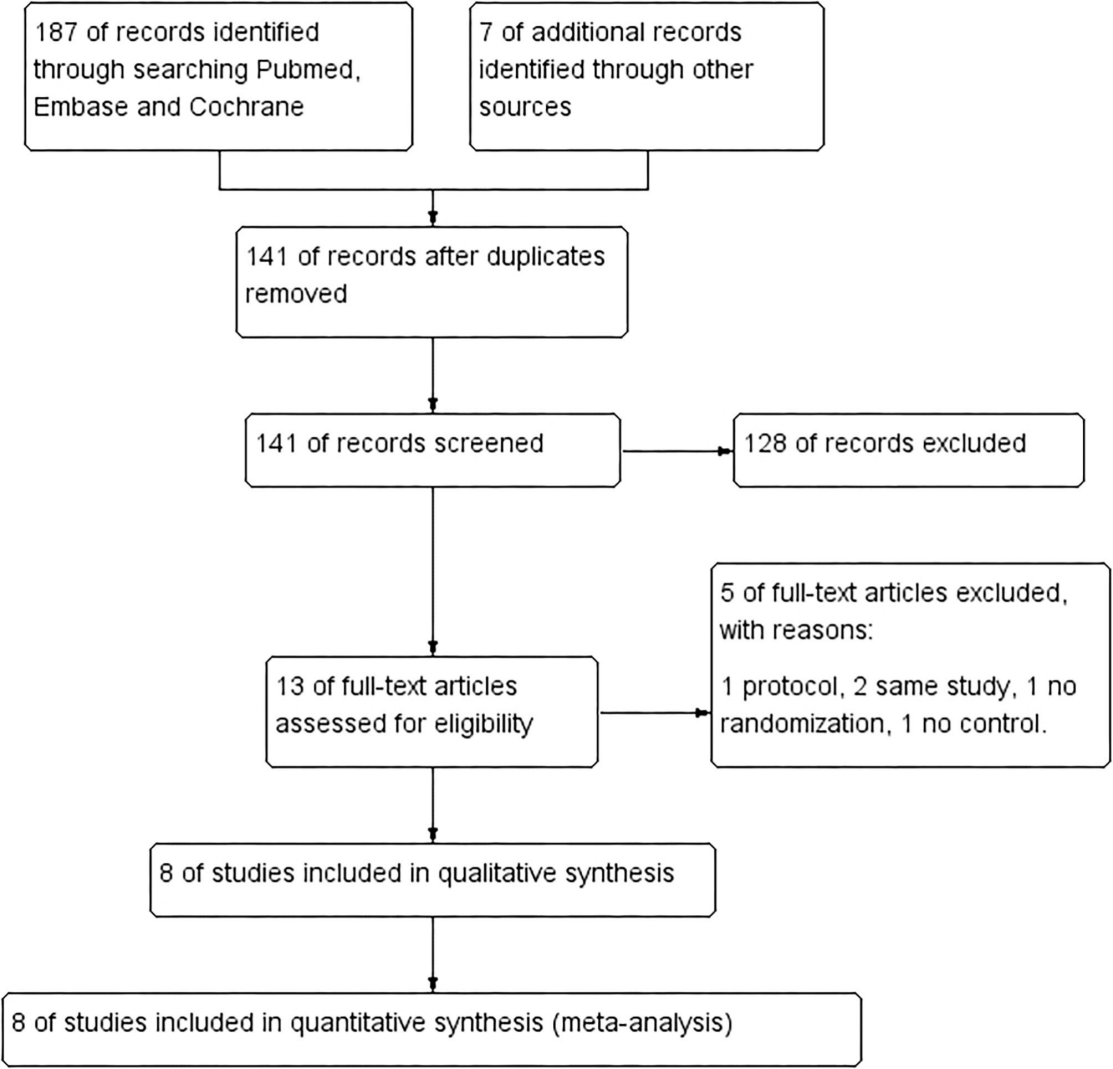
Flowchart of study selection.

We identified 194 citations in total by searching PubMed, Embase, and Cochrane database using our keywords. After removing 53 duplicates, we further excluded 128 citations by browsing titles and abstracts, and then we evaluated the remaining 13 citations with full text. Of the 13 full-text articles, five articles are excluded because of protocol, same study, no randomization, and no control. Finally, eight studies with 281 patients were included in our meta-analysis (flowchart in Figure 1 and search strategy details in the Supplementary Material) (8–11, 13–16).

### Characteristics of Eligible Studies

Of the included 281 patients, 149 (53.0%) were assigned to the SSNB group, and 135 (47.0%) were assigned to the control group. The follow-up period ranged from 4 to 12 wk. The largest RCT owns a sample number of 60 and the smallest of 10. The treatment for control group consists of two placebo controls and six active controls. The active control treatment included ultrasound treatment (16), intra-articular hyaluronic acid (HA) injection (15), intra-articular shoulder methyl prednisolone acetate injection (9), suprascapular nerve pulsed radiofrequency (11), botulinum toxin-A (BoNT-A) injection (10), and glenohumeral joint triamcinolone acetonide injection (13). The baseline details of the included studies are displayed in Table 1.

**Table 1:** Baseline characteristics of included studies.

**Study name**	**Journal**	**Total**	**SSNB**	**Control**	**Intervention**	**Way of control**	**Agents of SSNB**	**Agents of control**
Boonsong et al. (16)	J Med Assoc Thai	10	5	5	SSNB	Ultrasound Treatment	Lidocaine	Ultrasound Power
Adey-Wakeling et al. (8)	Stroke	64	32	32	SSNB	Placebo	Methylprednisolone +Bupivacaine Hydrochloride	Normal Saline
Kim and Kim (15)	Brain Neurorehabil	24	12	12	SSNB	Intra-Articular Hyaluronic Acid Injection	Lidocaine	Hyaluronic Acid
Sencan et al. (9)	Neurological Sciences	30	20	10	SSNB	Intraarticular Shoulder Injection	Bupivacaine	Methylprednisolone Acetate
Alanbay et al. (11)	Pain Physician	30	15	15	SSNB	Suprascapular nerve pulsed radiofrequency	Lidocaine	Pulsed Radiofrequency
Kasapoglu-Aksoy et al. (10)	Neurological Sciences	57	27	30	SSNB	Botulinum Toxin-A Injection	Lidocaine+Triamcinolone Hexacetonide	Botulinum Toxin-A
Terleme, et al. (14)	Neurological Sciences	30	20	10	SSNB	Placebo	Lidocaine	Lidocaine
Tubay et al. (13)	Turkiye Fiziksel Tip ve Rehabilitasyon Dergisi	36	18	18	SSNB	Glenohumeral Joint Injection	Prilocaine+Triamcinolone Acetonide	Prilocaine+Triamcinolone Acetonide

### Primary Endpoint of Visual Analog Scale

**Figure 2:**
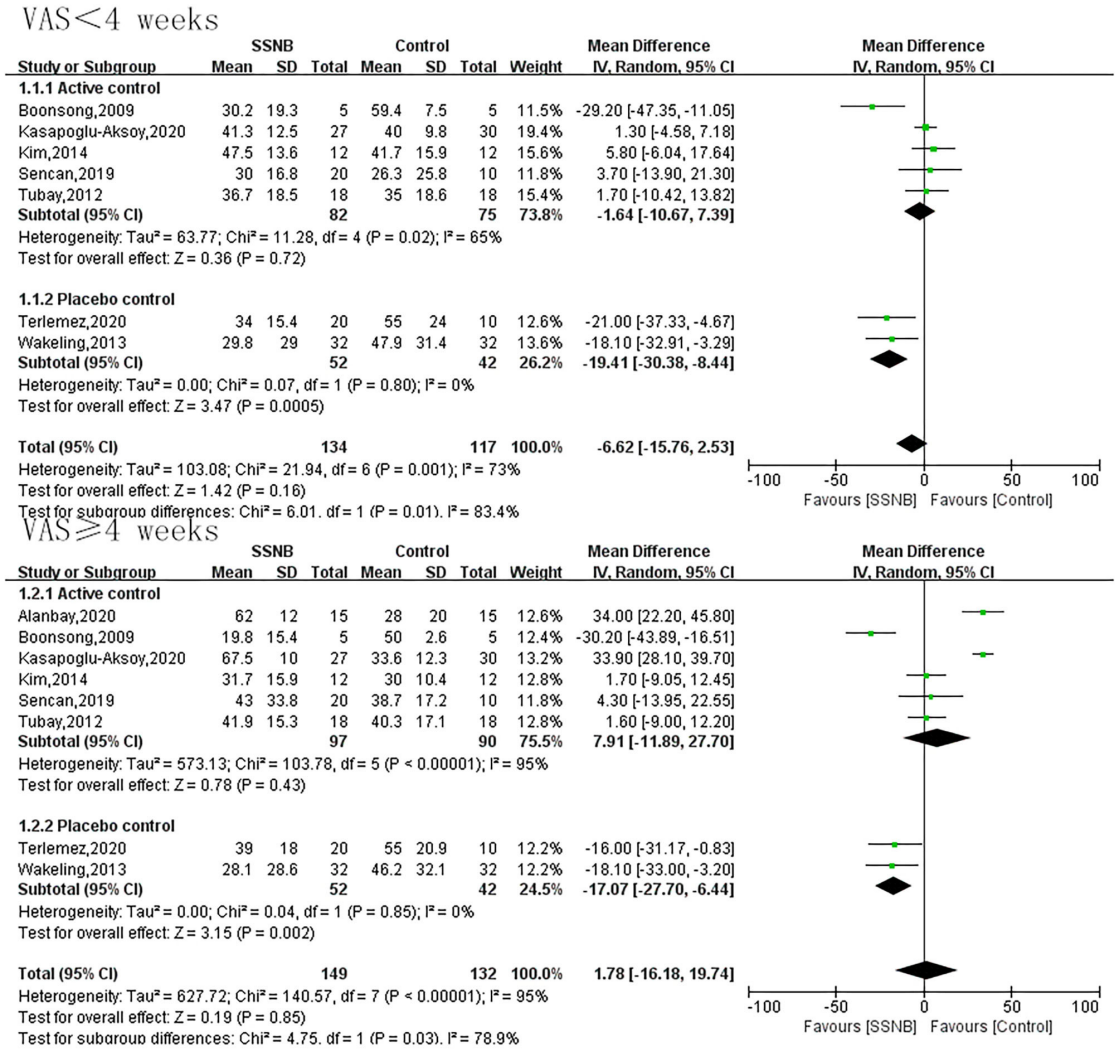
Forest plot of VAS between SSNB and other treatment.

VAS was an endpoint in all the eight included studies. For effects of SSNB on HSP within 4 wk, a total of 251 patients participated. For effects no less than 4 wk, 281 participated. As shown in Figure 2, there was no obvious difference between SSNB group and active control group regardless of the follow-up period (<4 or ≥4 wk), which were −1.64 (−10.67, 7.39; *p* = 0.72) and 7.91 (−11.89, 27.70; *p* = 0.43), but when compared with placebo, the SSNB showed obvious benefits than control group despite the follow-up period (<4 or ≥4 wk), and the corresponding mean differences (and 95% CI) were −19.41 (−30.38, −8.44; *p* = 0.0005) and −17.07 (−27.70, −6.44; *p* = 0.002) (Figure 2).

### Secondary Endpoints

**Figure 3:**
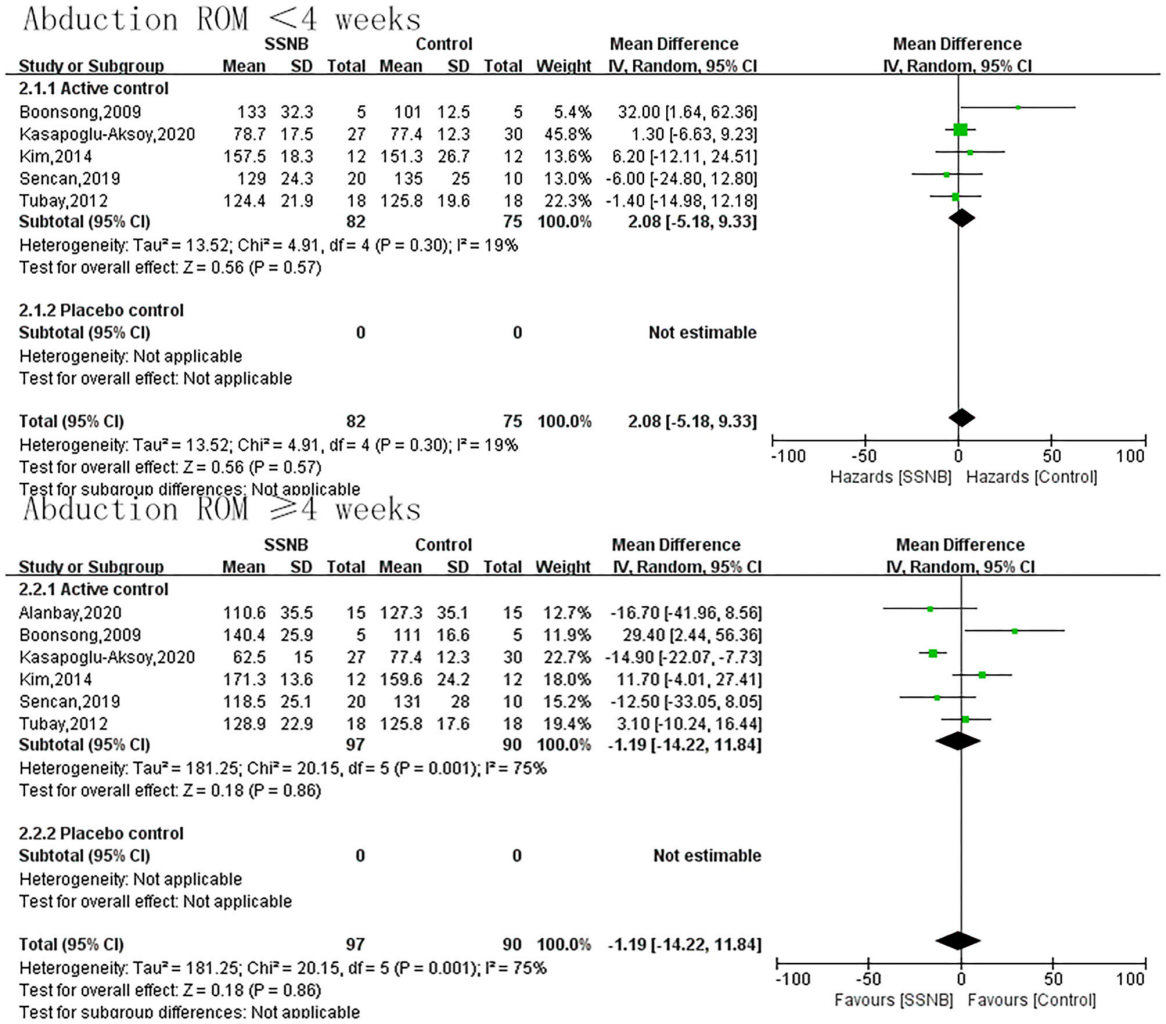
Forest plot of abduction ROM between SSNB and other treatment.

**Figure 4:**
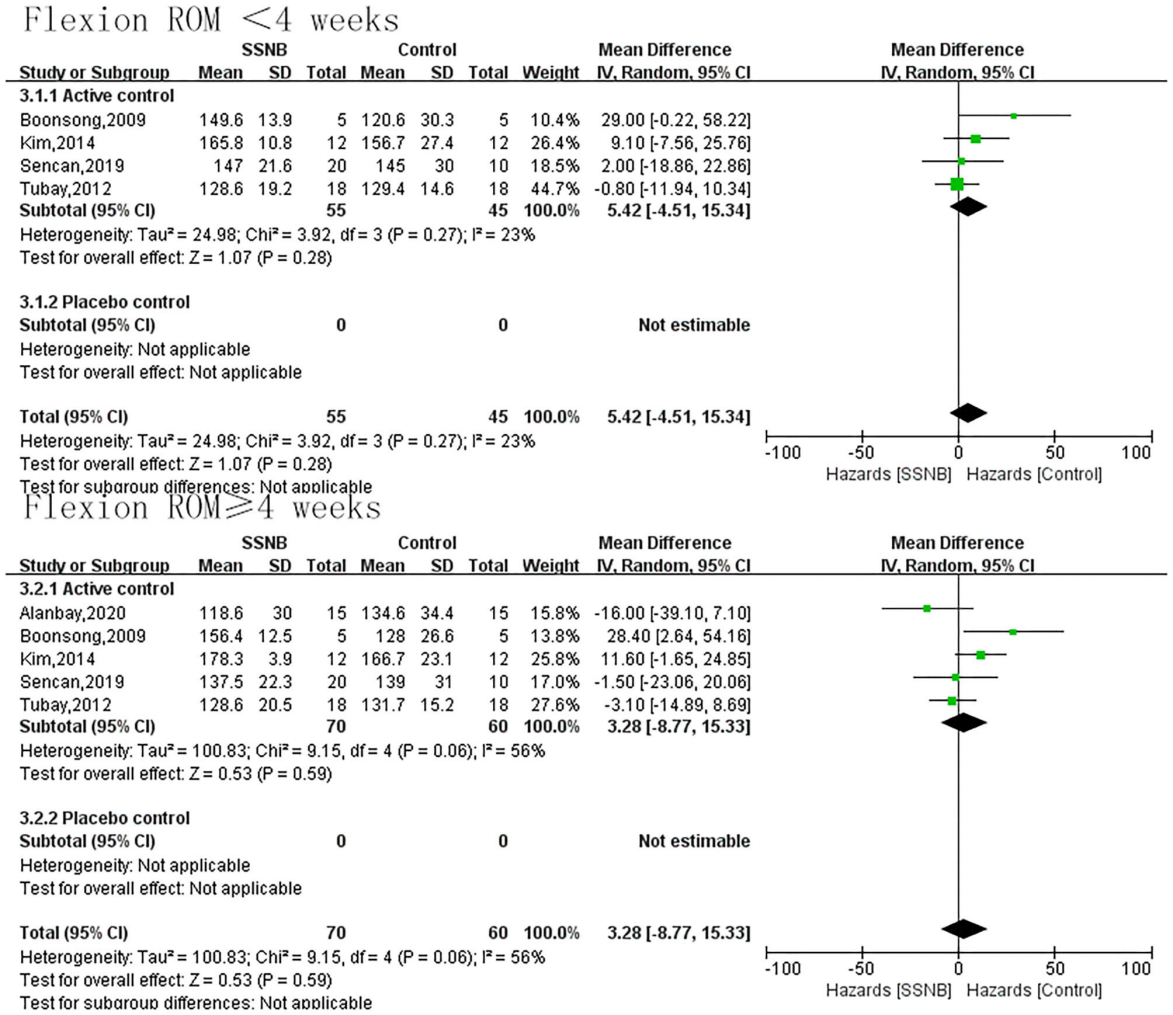
Forest plot of flexion ROM between SSNB and other treatment.

**Figure 5:**
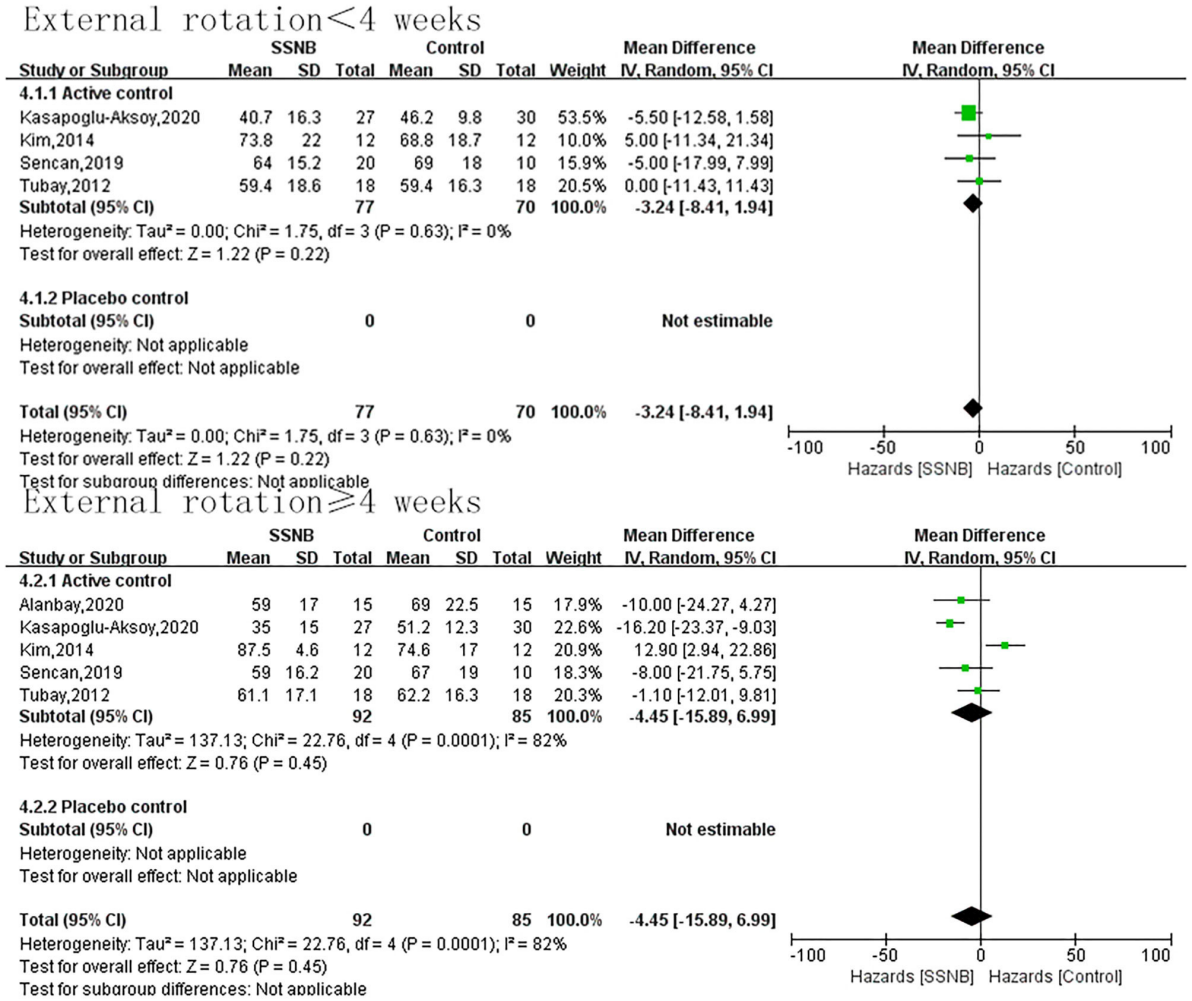
Forest plot of external rotation ROM between SSNB and other treatment.

**Figure 6:**
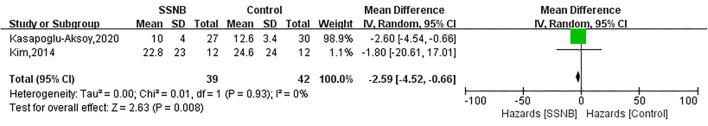
Forest plot of FMA between SSNB and other treatment.

The secondary endpoint we were interested in mainly included PROM of abduction, flexion, and external rotation. In addition, the indicator FMA reflecting the motor function was also a secondary outcome. For abduction, flexion, or external rotation ROM, similar with VAS, the difference between SSNB and active group was not statistically significant, which were 2.08 (−5.18, 9.33; *p* = 0.57), 5.42 (−4.51, 15.34; *p* = 0.28), and −3.24 (−8.41, 1.94; *p* = 0.22) in a follow-up less than 4 wk. When the follow-up period extended to over 4 wk, there were still no differences, and the mean differences (and 95% CI) were −1.19 (−14.22, 11.84; *p* = 0.86), −1.19 (−14.22, 11.84; *p* = 0.59), and −4.45 (−15.89, 6.99; *p* = 0.45) separately. For motor function of FMA, only two studies reported the outcome, and the FMA scores in SSNB group was −2.59 (−4.52, −0.66) less than those in active group (Figures 3–6).

### Publication Bias and Quality Assessment

A funnel plot was employed to test the publication bias, as shown in Supplementary Material. No obvious publication bias was observed. For quality assessment, we noticed that all the published articles had a high performance bias (Supplementary Material).

### Sensitivity Analysis

We performed sensitivity analysis for the primary endpoint VAS and found that our results were robust (Supplementary Material).

## Discussion

At present, SSNB is widely used in patients with chronic shoulder pain or frozen shoulder and gains excellent clinical effects (17, 18), but the application of SSNB in HSP patients is relatively rare. To the best of our knowledge, the present meta-analysis is the first one to investigate the effectiveness of SSNB vs. other treatments for HSP. In the analysis, we found that SSNB is an effective way to alleviate HSP at a longest follow-up period of 12 wk.

Suprascapular nerve is a mixed nerve fiber containing afferent and efferent content, and it originates from the upper trunk of the brachial plexus (C5, C6). The motor of supraspinatus and infraspinatus muscles is innervated by the suprascapular nerve, which is the basic of SSNB for the treatment of HSP (9, 19, 20). Given the different mechanisms of SSNB vs. other treatments, the treatment effects may differ. SSNB just temporarily blocked the suprascapular nerve; this may explain why the VAS failed to continue to decrease in the follow-up period more than 1 wk in the study by Adey-Wakeling et al. (8), but the VAS at weeks 1, 4, and 12 is much lower than baseline (about 30 vs. 69). In the study by Sencan et al. (9), the lowest VAS occurred in the second week after SSNB procedure, and the VAS at week 8 is similar to that at week 2. In our study, we synthesized data from eight studies involving 281 patients followed up for at least 4 wk and demonstrated that the VAS in SSNB is not higher than that in the other treatments. The reason why the pain relief still works after 4 wk may be that the patients move more after the SSNB, and this helps to relieve HSP; even though the pain relief from SSNB disappeared after 4 wk, the pain relief from increased movement is still sustained (21).

Compared with intra-articular shoulder injection (IAI), SSNB may be much safer; SSNB does not have side effects caused by steroids used in the IAI (9, 22). Some complications like intra-articular infection, which is common in the other treatments, we found no such complications reported in articles about SSNB. Besides effects in HSP relief, SSNB also has the advantage of cost-effectiveness; the current price of SSNB is much lower than that of other treatments like nerve pulsed radiofrequency treatment. It is also easy for the physical therapist to conduct the procedure.

In our analysis, we noticed that a combination of SSNB and other therapies may cause a better outcome. Sencan et al. (9) found that compared with SSNB or intra-articular shoulder injection (IAI), a combination of SSNB and IAI can reduce the VAS of HSP patients, although not different statistically, but the function of the shoulder improves significantly. Parashar et al. (23) divided 60 patients into three groups and found that SSNB in combination with non-invasive rehabilitation (NIR) is much more effective than either SSNB or NIR. One of the earliest studies about frozen shoulder also found that treatment with SSNB plus electroacupuncture is superior than any single one (24). Although the latter two studies focused on patients with chronic shoulder pain and frozen shoulder, it indicates that a combination of SSNB and other therapies may gain a better outcome.

The clinical benefits of injection guided by ultrasound or other equipment are still unknown. Compared with fluoroscopy-guided injection, the ultrasound-guided injection causes less harm to the therapist (9). In our clinical practice, the use of ultrasound can help us with a clear view of the tissues and may be much safer. In a cross-sectional study, the researchers found that compared with conservative treatment, the ultrasound-guided SSNB can obviously improve the pain relief, but it does not prove the role of ultrasound in the SSNB, as the control group received conservative treatment (25). Kang et al. (18) found that SSNB by fluoroscopy-guided anterior approach can reduce the dose of local anesthetics and avoid pneumothorax, indicating that ultrasound-guided injection may be a better method. However, a study published in 2020 found that ultrasound-guided SSNB did not improve the VAS or the shoulder function compared with landmark-guided SSNB in chronic shoulder pain patients (26). More RCTs are needed to verify the validity of ultrasound-guided SSNB.

## Limitations

The present meta-analysis has several limitations besides those inherent in the original studies. Firstly, some data in our study are transformed from the published articles, and this may cause the data to be not so accurate. Secondly, the control group patients received several kinds of treatment; this may introduce bias. Thirdly, our included studies reported no adverse events; it is impossible for us to investigate the safety of SSNB. Fourthly, the maximum follow-up period in our study is 12 wk; the efficacy of SSNB more than 3 months is unknown. Finally, the sample size in every study is small; this may introduce bias.

## Conclusion

SSNB is an effective way for HSP patients.

## Data Availability Statement

The original contributions presented in the study are included in the article/Supplementary Material, further inquiries can be directed to the corresponding author.

## Author Contributions

This study was designed by TZ. YH performed the study and wrote the manuscript. YW, XS, YL, and YY all participated in the study. All authors contributed to the article and approved the submitted version.

## Funding

This study was supported by the 2021 Outstanding Talents Project of Xicheng District, Beijing (Fund No. 202131).

## Conflict of Interest

The authors declare that the research was conducted in the absence of any commercial or financial relationships that could be construed as a potential conflict of interest.

## Publisher's Note

All claims expressed in this article are solely those of the authors and do not necessarily represent those of their affiliated organizations, or those of the publisher, the editors and the reviewers. Any product that may be evaluated in this article, or claim that may be made by its manufacturer, is not guaranteed or endorsed by the publisher.
