# Supplementary material for: Effectiveness of Suprascapular Nerve Block in the Treatment of Hemiplegic Shoulder Pain: A Systematic Review and Meta-Analysis
Source: Front Neurol. 2021 Oct 5;12:723664. doi: 10.3389/fneur.2021.723664 (PMC8523895; doi:10.3389/fneur.2021.723664)

**Cochrane search strategy**

Search Name:

Date Run: 02/05/2021 02:50:32

Comment:

ID Search Hits

#1 (hemiplegia):ti,ab,kw (Word variations have been searched) 2217

#2 MeSH descriptor: [Hemiplegia] explode all trees 748

#3 MeSH descriptor: [Hemiplegia] explode all trees 748

#4 (monoplegia):ti,ab,kw (Word variations have been searched) 10

#5 MeSH descriptor: [Paresis] explode all trees 899

#6 (paresis):ti,ab,kw (Word variations have been searched) 1969

#7 MeSH descriptor: [Paresis] explode all trees 899

#8 (hemiparesis):ti,ab,kw (Word variations have been searched) 1375

#9 MeSH descriptor: [Stroke] explode all trees 10259

#10 (cerebrovascular accident):ti,ab,kw (Word variations have been searched) 13585

#11 (stroke):ti,ab,kw (Word variations have been searched) 57918

#12 MeSH descriptor: [Basal Ganglia Hemorrhage] explode all trees 18

#13 MeSH descriptor: [Brain Ischemia] explode all trees 3701

#14 (brain ischemia):ti,ab,kw (Word variations have been searched) 6947

#15 MeSH descriptor: [Brain Infarction] explode all trees 1320

#16 (brain infarction):ti,ab,kw (Word variations have been searched) 4611

#17 MeSH descriptor: [Intracranial Hemorrhages] explode all trees 2019

#18 (intracranial hemorrhage):ti,ab,kw (Word variations have been searched) 3171

#19 MeSH descriptor: [Cerebral Hemorrhage] explode all trees 1025

#20 (cerebral hemorrhage):ti,ab,kw (Word variations have been searched) 4345

#21 MeSH descriptor: [Subarachnoid Hemorrhage] explode all trees 597

#22 (subarachnoid hemorrhage):ti,ab,kw (Word variations have been searched) 2057

#23 (poststroke):ti,ab,kw (Word variations have been searched) 4867

#24 (post-stroke):ti,ab,kw (Word variations have been searched) 4752

#25 #1 or #2 or #3 or #4 or #5 or #6 or #7 or #8 or #9 14174

#26 #10 or #11 or #12 or #13 or #14 or #15 or #16 or #17 or #18 65941

#27 #19 or #20 or #21 or #22 or #23 or #24 or #25 or #26 69736

#28 MeSH descriptor: [Shoulder Pain] explode all trees 963

#29 (shoulder pain):ti,ab,kw (Word variations have been searched) 7373

#30 (shoulders pain):ti,ab,kw (Word variations have been searched) 7373

#31 (shoulder pains):ti,ab,kw (Word variations have been searched) 7373

#32 (shoulders pains):ti,ab,kw (Word variations have been searched) 7373

#33 (upper limb spasticity):ti,ab,kw (Word variations have been searched) 919

#34 #28 or #29 or #30 or #31 or #32 or #33 8239

#35 #27 and #34 1209

#36 (interscalene):ti,ab,kw (Word variations have been searched) 824

#37 (Perineural):ti,ab,kw (Word variations have been searched) 862

#38 (bupivicaine):ti,ab,kw (Word variations have been searched) 212

#39 (ropivacaine):ti,ab,kw (Word variations have been searched) 6423

#40 (mepivicaine):ti,ab,kw (Word variations have been searched) 10

#41 (lidocaine):ti,ab,kw (Word variations have been searched) 13133

#42 MeSH descriptor: [Glucocorticoids] explode all trees 4613

#43 (glucocorticoid):ti,ab,kw (Word variations have been searched) 8885

#44 (glucocorticoids):ti,ab,kw (Word variations have been searched) 8886

#45 (nerve):ti,ab,kw (Word variations have been searched) 35146

#46 (nerves):ti,ab,kw (Word variations have been searched) 35149

#47 (block):ti,ab,kw (Word variations have been searched) 56985

#48 (blocks):ti,ab,kw (Word variations have been searched) 56951

#49 (Blockade):ti,ab,kw (Word variations have been searched) 14424

#50 #36 or #37 or #38 or #39 or #40 or #41 or #42 or #43 or #44 or #45 or #46 58558

#51 #47 or #48 or #49 65373

#52 #50 and #51 16682

#53 MeSH descriptor: [Nerve Block] explode all trees 4150

#54 (nerve block):ti,ab,kw (Word variations have been searched) 12256

#55 (nerves block):ti,ab,kw (Word variations have been searched) 12258

#56 (nerves blocks):ti,ab,kw (Word variations have been searched) 12257

#57 #52 or #53 or #54 or #55 or #56 16709

#58 #57 and #35 in Trials 99

**Pubmed search strategy**

Search number Query Sort By Filters Search Details Results Time

57 (((((((((((((((((((((((("Hemiplegia"[Mesh]) OR (hemiplegia[Title/Abstract])) OR (monoplegia[Title/Abstract])) OR ("Paresis"[Mesh])) OR (paresis[Title/Abstract])) OR (hemiparesis[Title/Abstract])) OR ("Stroke"[Mesh])) OR (cerebrovascular accident[Title/Abstract])) OR (stroke[Title/Abstract])) OR ("Basal Ganglia Hemorrhage"[Mesh])) OR (basal ganglia hemorrhage[Title/Abstract])) OR ("Brain Ischemia"[Mesh])) OR (brain ischemia[Title/Abstract])) OR ("Brain Infarction"[Mesh])) OR (brain infarction[Title/Abstract])) OR ("Intracranial Hemorrhages"[Mesh])) OR (intracranial hemorrhage[Title/Abstract])) OR ("Cerebral Hemorrhage"[Mesh])) OR (cerebral hemorrhage[Title/Abstract])) OR ("Subarachnoid Hemorrhage"[Mesh])) OR (subarachnoid hemorrhage[Title/Abstract])) OR (poststroke[Title/Abstract])) OR (post-stroke[Title/Abstract])) AND (((((("Shoulder Pain"[Mesh]) OR (shoulder pain[Title/Abstract])) OR (upper limb spasticity[Title/Abstract])) OR (shoulder pains[Title/Abstract])) OR (shoulders pain[Title/Abstract])) OR (shoulders pains[Title/Abstract]))) AND (((((((Blockade[Title/Abstract]) OR (blocks[Title/Abstract])) OR (block[Title/Abstract])) AND (((((((((((interscalene[Title/Abstract]) OR (nerve[Title/Abstract])) OR (nerves[Title/Abstract])) OR (Perineural[Title/Abstract])) OR (bupivicaine[Title/Abstract])) OR (ropivacaine[Title/Abstract])) OR (mepivicaine[Title/Abstract])) OR (lidocaine[Title/Abstract])) OR ("Glucocorticoids"[Mesh])) OR (glucocorticoid[Title/Abstract])) OR (glucocorticoids[Title/Abstract]))) OR (((Blockade[Title/Abstract]) OR (blocks[Title/Abstract])) OR (block[Title/Abstract]))) OR (nerve block[Title/Abstract])) OR (analgesia[Title/Abstract])) ("Hemiplegia"[MeSH Terms] OR "Hemiplegia"[Title/Abstract] OR "monoplegia"[Title/Abstract] OR "Paresis"[MeSH Terms] OR "Paresis"[Title/Abstract] OR "hemiparesis"[Title/Abstract] OR "Stroke"[MeSH Terms] OR "cerebrovascular accident"[Title/Abstract] OR "Stroke"[Title/Abstract] OR "Basal Ganglia Hemorrhage"[MeSH Terms] OR "Basal Ganglia Hemorrhage"[Title/Abstract] OR "Brain Ischemia"[MeSH Terms] OR "Brain Ischemia"[Title/Abstract] OR "Brain Infarction"[MeSH Terms] OR "Brain Infarction"[Title/Abstract] OR "Intracranial Hemorrhages"[MeSH Terms] OR "intracranial hemorrhage"[Title/Abstract] OR "Cerebral Hemorrhage"[MeSH Terms] OR "Cerebral Hemorrhage"[Title/Abstract] OR "Subarachnoid Hemorrhage"[MeSH Terms] OR "Subarachnoid Hemorrhage"[Title/Abstract] OR "poststroke"[Title/Abstract] OR "post-stroke"[Title/Abstract]) AND ("Shoulder Pain"[MeSH Terms] OR "Shoulder Pain"[Title/Abstract] OR "upper limb spasticity"[Title/Abstract] OR "shoulder pains"[Title/Abstract] OR "shoulders pain"[Title/Abstract] OR (("shoulder"[MeSH Terms] OR "shoulder"[All Fields] OR "shoulders"[All Fields] OR "shoulder s"[All Fields]) AND "pains"[Title/Abstract])) AND ((("Blockade"[Title/Abstract] OR "blocks"[Title/Abstract] OR "block"[Title/Abstract]) AND ("interscalene"[Title/Abstract] OR "nerve"[Title/Abstract] OR "nerves"[Title/Abstract] OR "Perineural"[Title/Abstract] OR "bupivicaine"[Title/Abstract] OR "ropivacaine"[Title/Abstract] OR "mepivicaine"[Title/Abstract] OR "lidocaine"[Title/Abstract] OR "Glucocorticoids"[MeSH Terms] OR "glucocorticoid"[Title/Abstract] OR "Glucocorticoids"[Title/Abstract])) OR ("Blockade"[Title/Abstract] OR "blocks"[Title/Abstract] OR "block"[Title/Abstract]) OR "nerve block"[Title/Abstract] OR "analgesia"[Title/Abstract]) 36 2:40:15

56 ((((((Blockade[Title/Abstract]) OR (blocks[Title/Abstract])) OR (block[Title/Abstract])) AND (((((((((((interscalene[Title/Abstract]) OR (nerve[Title/Abstract])) OR (nerves[Title/Abstract])) OR (Perineural[Title/Abstract])) OR (bupivicaine[Title/Abstract])) OR (ropivacaine[Title/Abstract])) OR (mepivicaine[Title/Abstract])) OR (lidocaine[Title/Abstract])) OR ("Glucocorticoids"[Mesh])) OR (glucocorticoid[Title/Abstract])) OR (glucocorticoids[Title/Abstract]))) OR (((Blockade[Title/Abstract]) OR (blocks[Title/Abstract])) OR (block[Title/Abstract]))) OR (nerve block[Title/Abstract])) OR (analgesia[Title/Abstract]) (("Blockade"[Title/Abstract] OR "blocks"[Title/Abstract] OR "block"[Title/Abstract]) AND ("interscalene"[Title/Abstract] OR "nerve"[Title/Abstract] OR "nerves"[Title/Abstract] OR "Perineural"[Title/Abstract] OR "bupivicaine"[Title/Abstract] OR "ropivacaine"[Title/Abstract] OR "mepivicaine"[Title/Abstract] OR "lidocaine"[Title/Abstract] OR "Glucocorticoids"[MeSH Terms] OR "glucocorticoid"[Title/Abstract] OR "Glucocorticoids"[Title/Abstract])) OR ("Blockade"[Title/Abstract] OR "blocks"[Title/Abstract] OR "block"[Title/Abstract]) OR "nerve block"[Title/Abstract] OR "analgesia"[Title/Abstract] 506,234 2:31:52

55 (((Blockade[Title/Abstract]) OR (blocks[Title/Abstract])) OR (block[Title/Abstract])) AND (((((((((((interscalene[Title/Abstract]) OR (nerve[Title/Abstract])) OR (nerves[Title/Abstract])) OR (Perineural[Title/Abstract])) OR (bupivicaine[Title/Abstract])) OR (ropivacaine[Title/Abstract])) OR (mepivicaine[Title/Abstract])) OR (lidocaine[Title/Abstract])) OR ("Glucocorticoids"[Mesh])) OR (glucocorticoid[Title/Abstract])) OR (glucocorticoids[Title/Abstract])) ("Blockade"[Title/Abstract] OR "blocks"[Title/Abstract] OR "block"[Title/Abstract]) AND ("interscalene"[Title/Abstract] OR "nerve"[Title/Abstract] OR "nerves"[Title/Abstract] OR "Perineural"[Title/Abstract] OR "bupivicaine"[Title/Abstract] OR "ropivacaine"[Title/Abstract] OR "mepivicaine"[Title/Abstract] OR "lidocaine"[Title/Abstract] OR "Glucocorticoids"[MeSH Terms] OR "glucocorticoid"[Title/Abstract] OR "Glucocorticoids"[Title/Abstract]) 38,366 2:31:27

54 ((((((((((interscalene[Title/Abstract]) OR (nerve[Title/Abstract])) OR (nerves[Title/Abstract])) OR (Perineural[Title/Abstract])) OR (bupivicaine[Title/Abstract])) OR (ropivacaine[Title/Abstract])) OR (mepivicaine[Title/Abstract])) OR (lidocaine[Title/Abstract])) OR ("Glucocorticoids"[Mesh])) OR (glucocorticoid[Title/Abstract])) OR (glucocorticoids[Title/Abstract]) "interscalene"[Title/Abstract] OR "nerve"[Title/Abstract] OR "nerves"[Title/Abstract] OR "Perineural"[Title/Abstract] OR "bupivicaine"[Title/Abstract] OR "ropivacaine"[Title/Abstract] OR "mepivicaine"[Title/Abstract] OR "lidocaine"[Title/Abstract] OR "Glucocorticoids"[MeSH Terms] OR "glucocorticoid"[Title/Abstract] OR "Glucocorticoids"[Title/Abstract] 572,477 2:29:25

53 ((((((((((((((((((((((("Hemiplegia"[Mesh]) OR (hemiplegia[Title/Abstract])) OR (monoplegia[Title/Abstract])) OR ("Paresis"[Mesh])) OR (paresis[Title/Abstract])) OR (hemiparesis[Title/Abstract])) OR ("Stroke"[Mesh])) OR (cerebrovascular accident[Title/Abstract])) OR (stroke[Title/Abstract])) OR ("Basal Ganglia Hemorrhage"[Mesh])) OR (basal ganglia hemorrhage[Title/Abstract])) OR ("Brain Ischemia"[Mesh])) OR (brain ischemia[Title/Abstract])) OR ("Brain Infarction"[Mesh])) OR (brain infarction[Title/Abstract])) OR ("Intracranial Hemorrhages"[Mesh])) OR (intracranial hemorrhage[Title/Abstract])) OR ("Cerebral Hemorrhage"[Mesh])) OR (cerebral hemorrhage[Title/Abstract])) OR ("Subarachnoid Hemorrhage"[Mesh])) OR (subarachnoid hemorrhage[Title/Abstract])) OR (poststroke[Title/Abstract])) OR (post-stroke[Title/Abstract])) AND (((((("Shoulder Pain"[Mesh]) OR (shoulder pain[Title/Abstract])) OR (upper limb spasticity[Title/Abstract])) OR (shoulder pains[Title/Abstract])) OR (shoulders pain[Title/Abstract])) OR (shoulders pains[Title/Abstract])) ("Hemiplegia"[MeSH Terms] OR "Hemiplegia"[Title/Abstract] OR "monoplegia"[Title/Abstract] OR "Paresis"[MeSH Terms] OR "Paresis"[Title/Abstract] OR "hemiparesis"[Title/Abstract] OR "Stroke"[MeSH Terms] OR "cerebrovascular accident"[Title/Abstract] OR "Stroke"[Title/Abstract] OR "Basal Ganglia Hemorrhage"[MeSH Terms] OR "Basal Ganglia Hemorrhage"[Title/Abstract] OR "Brain Ischemia"[MeSH Terms] OR "Brain Ischemia"[Title/Abstract] OR "Brain Infarction"[MeSH Terms] OR "Brain Infarction"[Title/Abstract] OR "Intracranial Hemorrhages"[MeSH Terms] OR "intracranial hemorrhage"[Title/Abstract] OR "Cerebral Hemorrhage"[MeSH Terms] OR "Cerebral Hemorrhage"[Title/Abstract] OR "Subarachnoid Hemorrhage"[MeSH Terms] OR "Subarachnoid Hemorrhage"[Title/Abstract] OR "poststroke"[Title/Abstract] OR "post-stroke"[Title/Abstract]) AND ("Shoulder Pain"[MeSH Terms] OR "Shoulder Pain"[Title/Abstract] OR "upper limb spasticity"[Title/Abstract] OR "shoulder pains"[Title/Abstract] OR "shoulders pain"[Title/Abstract] OR (("shoulder"[MeSH Terms] OR "shoulder"[All Fields] OR "shoulders"[All Fields] OR "shoulder s"[All Fields]) AND "pains"[Title/Abstract])) 739 2:28:05

31 ((((("Shoulder Pain"[Mesh]) OR (shoulder pain[Title/Abstract])) OR (upper limb spasticity[Title/Abstract])) OR (shoulder pains[Title/Abstract])) OR (shoulders pain[Title/Abstract])) OR (shoulders pains[Title/Abstract]) "Shoulder Pain"[MeSH Terms] OR "Shoulder Pain"[Title/Abstract] OR "upper limb spasticity"[Title/Abstract] OR "shoulder pains"[Title/Abstract] OR "shoulders pain"[Title/Abstract] OR (("shoulder"[MeSH Terms] OR "shoulder"[All Fields] OR "shoulders"[All Fields] OR "shoulder s"[All Fields]) AND "pains"[Title/Abstract]) 10,243 2:27:25

52 (((((((((((((((((((((("Hemiplegia"[Mesh]) OR (hemiplegia[Title/Abstract])) OR (monoplegia[Title/Abstract])) OR ("Paresis"[Mesh])) OR (paresis[Title/Abstract])) OR (hemiparesis[Title/Abstract])) OR ("Stroke"[Mesh])) OR (cerebrovascular accident[Title/Abstract])) OR (stroke[Title/Abstract])) OR ("Basal Ganglia Hemorrhage"[Mesh])) OR (basal ganglia hemorrhage[Title/Abstract])) OR ("Brain Ischemia"[Mesh])) OR (brain ischemia[Title/Abstract])) OR ("Brain Infarction"[Mesh])) OR (brain infarction[Title/Abstract])) OR ("Intracranial Hemorrhages"[Mesh])) OR (intracranial hemorrhage[Title/Abstract])) OR ("Cerebral Hemorrhage"[Mesh])) OR (cerebral hemorrhage[Title/Abstract])) OR ("Subarachnoid Hemorrhage"[Mesh])) OR (subarachnoid hemorrhage[Title/Abstract])) OR (poststroke[Title/Abstract])) OR (post-stroke[Title/Abstract]) "Hemiplegia"[MeSH Terms] OR "Hemiplegia"[Title/Abstract] OR "monoplegia"[Title/Abstract] OR "Paresis"[MeSH Terms] OR "Paresis"[Title/Abstract] OR "hemiparesis"[Title/Abstract] OR "Stroke"[MeSH Terms] OR "cerebrovascular accident"[Title/Abstract] OR "Stroke"[Title/Abstract] OR "Basal Ganglia Hemorrhage"[MeSH Terms] OR "Basal Ganglia Hemorrhage"[Title/Abstract] OR "Brain Ischemia"[MeSH Terms] OR "Brain Ischemia"[Title/Abstract] OR "Brain Infarction"[MeSH Terms] OR "Brain Infarction"[Title/Abstract] OR "Intracranial Hemorrhages"[MeSH Terms] OR "intracranial hemorrhage"[Title/Abstract] OR "Cerebral Hemorrhage"[MeSH Terms] OR "Cerebral Hemorrhage"[Title/Abstract] OR "Subarachnoid Hemorrhage"[MeSH Terms] OR "Subarachnoid Hemorrhage"[Title/Abstract] OR "poststroke"[Title/Abstract] OR "post-stroke"[Title/Abstract] 436,677 2:26:29

51 analgesia[Title/Abstract] "analgesia"[Title/Abstract] 68,684 23:17:01

50 nerve block[Title/Abstract] "nerve block"[Title/Abstract] 9,090 23:16:43

49 "Nerve Block"[Mesh] Most Recent "Nerve Block"[MeSH Terms] 23,181 23:16:27

48 ((Blockade[Title/Abstract]) OR (blocks[Title/Abstract])) OR (block[Title/Abstract]) "Blockade"[Title/Abstract] OR "blocks"[Title/Abstract] OR "block"[Title/Abstract] 449,327 23:15:59

47 Blockade[Title/Abstract] "Blockade"[Title/Abstract] 120,074 23:15:45

46 blocks[Title/Abstract] "blocks"[Title/Abstract] 122,410 23:15:25

45 block[Title/Abstract] "block"[Title/Abstract] 243,466 23:15:06

43 glucocorticoids[Title/Abstract] "glucocorticoids"[Title/Abstract] 35,731 23:12:15

42 glucocorticoid[Title/Abstract] "glucocorticoid"[Title/Abstract] 51,870 23:12:06

41 "Glucocorticoids"[Mesh] Most Recent "Glucocorticoids"[MeSH Terms] 65,932 23:11:55

40 lidocaine[Title/Abstract] "lidocaine"[Title/Abstract] 22,710 23:08:17

39 mepivicaine[Title/Abstract] "mepivicaine"[Title/Abstract] 20 23:08:03

38 ropivacaine[Title/Abstract] "ropivacaine"[Title/Abstract] 4,772 23:07:46

37 bupivicaine[Title/Abstract] "bupivicaine"[Title/Abstract] 168 23:07:30

36 Perineural[Title/Abstract] "Perineural"[Title/Abstract] 8,074 23:06:56

35 nerves[Title/Abstract] "nerves"[Title/Abstract] 99,053 23:06:36

34 nerve[Title/Abstract] "nerve"[Title/Abstract] 385,416 23:06:20

33 interscalene[Title/Abstract] "interscalene"[Title/Abstract] 1,351 23:06:03

30 shoulders pains[Title/Abstract] ("shoulder"[MeSH Terms] OR "shoulder"[All Fields] OR "shoulders"[All Fields] OR "shoulder s"[All Fields]) AND "pains"[Title/Abstract] 249 23:03:48

29 shoulders pain[Title/Abstract] "shoulders pain"[Title/Abstract] 20 23:03:33

28 shoulder pains[Title/Abstract] "shoulder pains"[Title/Abstract] 44 23:03:20

27 upper limb spasticity[Title/Abstract] "upper limb spasticity"[Title/Abstract] 311 23:01:24

26 shoulder pain[Title/Abstract] "shoulder pain"[Title/Abstract] 7,377 23:00:49

25 "Shoulder Pain"[Mesh] Most Recent "Shoulder Pain"[MeSH Terms] 5,089 23:00:32

23 post-stroke[Title/Abstract] "post-stroke"[Title/Abstract] 10,495 22:57:01

22 poststroke[Title/Abstract] "poststroke"[Title/Abstract] 14,433 22:56:43

21 subarachnoid hemorrhage[Title/Abstract] "subarachnoid hemorrhage"[Title/Abstract] 21,946 22:56:00

20 "Subarachnoid Hemorrhage"[Mesh] Most Recent "Subarachnoid Hemorrhage"[MeSH Terms] 21,885 22:55:49

19 cerebral hemorrhage[Title/Abstract] "cerebral hemorrhage"[Title/Abstract] 7,411 22:55:20

18 "Cerebral Hemorrhage"[Mesh] Most Recent "Cerebral Hemorrhage"[MeSH Terms] 34,763 22:55:06

17 intracranial hemorrhage[Title/Abstract] "intracranial hemorrhage"[Title/Abstract] 10,496 22:54:40

16 "Intracranial Hemorrhages"[Mesh] Most Recent "Intracranial Hemorrhages"[MeSH Terms] 73,279 22:54:28

15 brain infarction[Title/Abstract] "brain infarction"[Title/Abstract] 2,530 22:53:56

14 "Brain Infarction"[Mesh] Most Recent "Brain Infarction"[MeSH Terms] 39,119 22:53:43

13 brain ischemia[Title/Abstract] "brain ischemia"[Title/Abstract] 5,708 22:53:21

12 "Brain Ischemia"[Mesh] Most Recent "Brain Ischemia"[MeSH Terms] 112,561 22:53:00

11 basal ganglia hemorrhage[Title/Abstract] "basal ganglia hemorrhage"[Title/Abstract] 160 22:52:23

10 "Basal Ganglia Hemorrhage"[Mesh] Most Recent "Basal Ganglia Hemorrhage"[MeSH Terms] 345 22:52:13

9 stroke[Title/Abstract] "stroke"[Title/Abstract] 260,821 22:51:44

8 cerebrovascular accident[Title/Abstract] "cerebrovascular accident"[Title/Abstract] 4,794 22:51:20

7 "Stroke"[Mesh] Most Recent "Stroke"[MeSH Terms] 142,907 22:50:59

6 hemiparesis[Title/Abstract] "hemiparesis"[Title/Abstract] 10,953 22:50:23

5 paresis[Title/Abstract] "paresis"[Title/Abstract] 10,848 22:49:44

4 "Paresis"[Mesh] Most Recent "Paresis"[MeSH Terms] 8,292 22:49:30

3 monoplegia[Title/Abstract] "monoplegia"[Title/Abstract] 211 22:48:58

2 hemiplegia[Title/Abstract] "hemiplegia"[Title/Abstract] 8,989 22:48:14

1 "Hemiplegia"[Mesh] Most Recent "Hemiplegia"[MeSH Terms] 11,635 22:47:57

Funnel plot


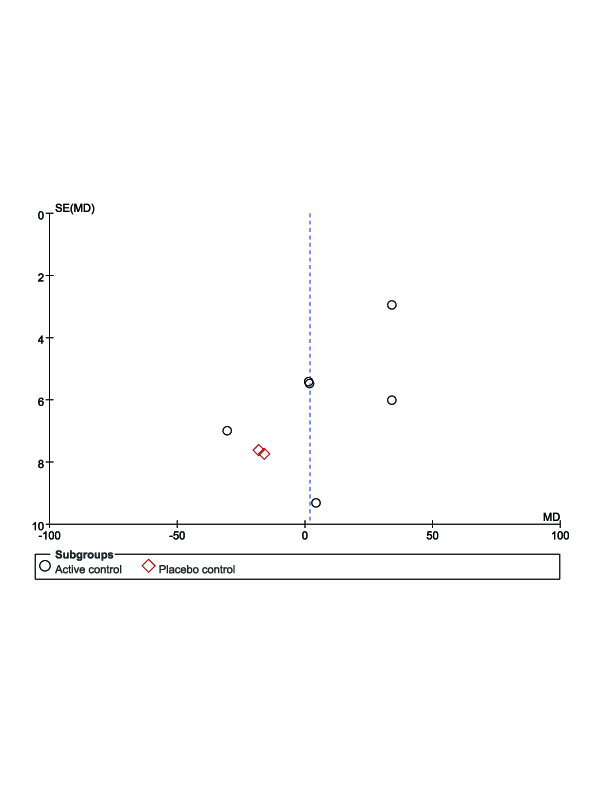


Risk of bias (According to Cochrane handbook)


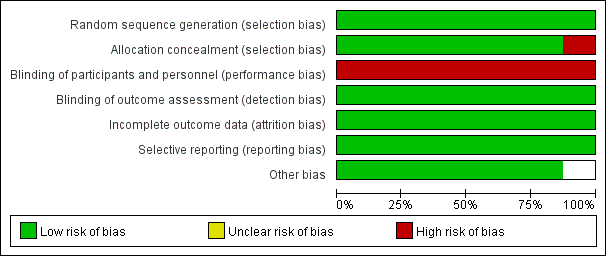


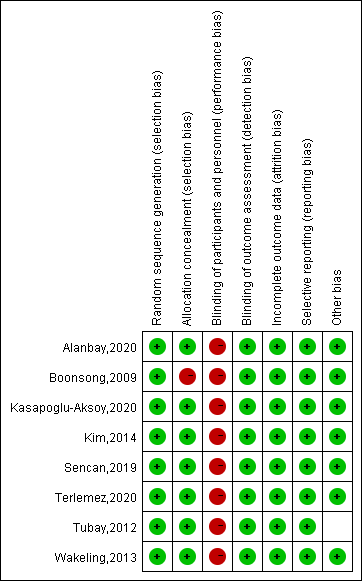


Influence analysis


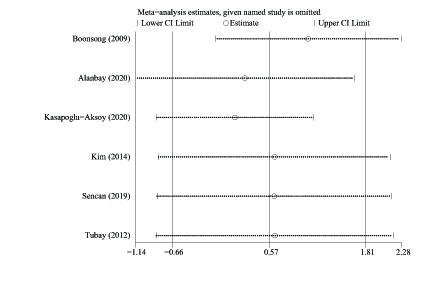

Supplement: Supplementary file 1 [file Data_Sheet_1.doc]
